# Genome-Wide Characterization and Transcriptional Profiling of the WRKY Gene Family During Heartwood Formation in *Dalbergia odorifera*

**DOI:** 10.3390/genes17040386

**Published:** 2026-03-28

**Authors:** Ruoke Ma, Yueyao Xu, Heng Liu, Qianying Wei, Jia Luo, Boling Liu, Yunlin Fu

**Affiliations:** 1 School of Life Sciences, Qufu Normal University, Qufu 273165, China; maruoke@163.com (R.M.); xuyy0506@163.com (Y.X.); wei13561276080@163.com (Q.W.); 2 Shandong Key Laboratory of Wetland Ecology and Biodiversity Conservation in the Lower Yellow River, Qufu 273165, China; 3 Experimental Center of Tropical Forestry, Chinese Academy of Forestry, Pingxiang 532600, China; lljheng@126.com; 4 Chongqing Forestry Investment Development Co., Ltd., Chongqing 400000, China; 18284905882@163.com; 5 Guangxi Key Laboratory of Forest Ecology and Conservation, Key Laboratory of National Forestry and Grassland Administration on Cultivation of Fast-Growing Timber in Central South China, College of Forestry, Guangxi University, Nanning 530004, China

**Keywords:** WRKY transcription factors, *Dalbergia odorifera*, heartwood formation, comparative phylogenetic analysis, differential expression analysis

## Abstract

Background: The WRKY transcription factor family represents one of the most crucial transcription factor families in plants, regulating diverse physiological processes. The heartwood of *Dalbergia odorifera* is a prized material for both high-quality rosewood and traditional medicinal applications, exhibiting exceptional economic value. However, the roles of WRKY transcription factors in the growth and development of *D. odorifera*, particularly their correlation with heartwood formation, remain unexplored. Methods: WRKY transcription factors were identified through bioinformatics analysis using the published genome data of *D. odorifera*. Phylogenetic comparative analysis was performed based on the *Arabidopsis* classification system. Collinearity analysis was conducted to investigate the evolutionary dynamics and expansion mechanisms of the WRKY gene family, and differential expression analysis was performed across tissues. Results: A total of 94 WRKY genes were unevenly distributed across 10 chromosomes and systematically designated as *DodWRKY1* to *DodWRKY94* according to their chromosomal positions. The WRKY family was classified into three major clades (Groups I, II, and III), with Group II further subdivided into five subgroups (IIa–IIe). Purifying selection served as the primary force shaping the WRKY family, with whole-genome or segmental duplication acting as the dominant expansion mechanism; these duplication events contributed to functional divergence, whereas genes within the same subgroup retained conserved structural features and motif compositions. *DodWRKY14* (subgroup IIb) and *DodWRKY*58/68 (subgroup IIc) were highly expressed in the transition zone, suggesting a potential involvement in heartwood formation. Conclusions: This study provides a comprehensive characterization of the *DodWRKY* family and identifies candidate genes associated with heartwood formation, thereby establishing a foundation for further investigation into the molecular mechanisms underlying heartwood development.

## 1. Introduction

*Dalbergia odorifera* T. Chen, commonly known in China as Hainan Huanghuali, is a semi-deciduous tree belonging to the Fabaceae family and the genus *Dalbergia*. Renowned for the exceptional quality of its heartwood, this species exhibits outstanding decay resistance, high density, aesthetically distinctive grain patterns and colors, and characteristic aromatic volatiles [1,2]. These attributes have made its heartwood a prized material for high-end rosewood furniture manufacturing and artistic carving, establishing it as one of the most economically valuable species under current international rosewood classification standards [3]. Additionally, the heartwood is rich in bioactive medicinal compounds, forming a traditional Chinese herbal medicine known as “Jiangxiang,” which demonstrates therapeutic effects such as improving cardiovascular function and anti-rheumatic properties [4]. Owing to the economic and cultural significance of the species, research has focused on “studying the molecular mechanisms underlying heartwood development”. Native to the tropical monsoon climate region of Hainan Island, China, the cultivation of *D. odorifera* has expanded in recent years to subtropical regions such as Fujian, Guangdong, Guangxi, Yunnan, and Zhejiang, driven by surging market demand, resulting in large-scale plantations that continue to grow in area [5]. However, its heartwood formation cycle is lengthy, and mature heartwood occupies a low proportion within the trunk’s cross-sectional area, making the regulation of heartwood development a critical factor in enhancing the species’ utilization value [6]. With the release of its genomic data [7], identifying molecular mechanisms that regulate xylem heartwood formation is currently a key research priority.

Transcription factors (TFs) are protein molecules that bind specifically to cis-acting elements in the promoter regions of eukaryotic genes. Through interactions with these elements and other related proteins, TFs activate or repress transcription, ensuring the spatiotemporally ordered expression of target genes [8]. Based on the similarity of their binding sites, TFs are classified into distinct families, such as AP2/EREBP, bHLH, HB, MYB, and WRKY. Among these, the WRKY gene family is one of the largest TF families in plants and plays pivotal roles in regulating plant growth, development, and stress responses [4]. WRKY transcription factors are characterized by a highly conserved WRKYGQK amino acid sequence at the N-terminus and a specific zinc finger motif (C_2_H_2_ or C_2_HC) at the C-terminus. The WRKY family is divided into three major groups: Group I, Group II, and Group III. Group I contains two WRKY heptapeptide conserved sequences and a C_2_H_2_-type zinc finger structure. Group II possesses one WRKY heptapeptide conserved sequence and a C_2_H_2_-type zinc finger structure and is further subdivided into five subgroups (IIa, IIb, IIc, IId, and IIe) based on amino acid sequence variations. Group III features one WRKY heptapeptide conserved sequence and a C_2_HC-type zinc finger structure [8]. In higher plants, the WRKY gene family is predominantly composed of Group II members, with subgroup IIc being the most abundant.

With the completion of genome sequencing for numerous higher plants, an increasing number of WRKY transcription factors (TFs) have been identified and functionally characterized across diverse plant species, including the model plant *Arabidopsis thaliana*, rice (*Oryza sativa* L.) [9], sunflower (*Helianthus annuus* L.) [10], pea (*Pisum sativum* L.) [11], and poplar (*Populus* trichocarpa Torr. & A.Gray ex. Hook.) [12]. Studies have demonstrated that WRKY TFs play critical roles in regulating plant growth and developmental processes [13]. For example, the rice *OsWRKY78* modulates stem elongation and seed development [14], while the *AtWRKY12/AtWRKY13* gene pair in *Arabidopsis* antagonistically regulates the gibberellin signaling pathway to precisely control flowering time under short-day conditions [15]. WRKY TFs also exert significant regulatory effects on plant secondary metabolism [16]. Overexpression of *MdWRKY11* in apple (*Malus domestica* (Suckow) Borkh.) callus tissues, for instance, markedly activates the expression of key genes (*MdF3H*, *MdFLS*, *MdDFR*, *MdANS*, and *MdUFGT*) in the flavonoid biosynthesis pathway, thereby promoting the accumulation of anthocyanins and flavonoids [17]. Furthermore, WRKY TFs are central to plant stress responses, mediating adaptation to diverse biotic and abiotic stresses such as drought, salinity, and pathogen infection [8]. Research indicates that the *ZmWRKY79* TF enhances drought tolerance in maize by upregulating the ABA biosynthesis pathway [18], whereas *SmWRKY11* positively regulates salt tolerance in eggplant (*Solanum melongena* L.) [19]. In summary, WRKY TFs serve as pivotal components in the regulatory networks governing plant development, metabolism, and stress adaptation. However, the number, classification, and functional roles of WRKY TFs in the precious tree species *D. odorifera* remain entirely unknown.

This study utilizes bioinformatic tools to systematically identify and analyze the WRKY transcription factor (TF) family in the *D. odorifera* genome. The research aims to address the following: (1) Characterization of physicochemical properties and conserved domains of *D. odorifera* WRKY proteins; (2) Evolutionary relationships and functional prediction of WRKY proteins; (3) Identification of WRKY genes associated with xylem heartwood formation based on their tissue-specific expression patterns. By elucidating the structural features, evolutionary relationships, and functional roles of WRKY genes in *D. odorifera*, this study seeks to uncover molecular mechanisms regulating heartwood formation. The findings are expected to provide theoretical insights and practical references for the artificial cultivation of *D. odorifera* and the scientific regulation of heartwood development.

## 2. Materials and Methods

### 2.1. Identification and Screening of WRKY Transcription Factor Family in the D. odorifera Genome

The genome data of *D. odorifera* were downloaded from the website http://gigadb.org/dataset/100760 (accessed on 24 August 2025). To screen WRKY genes, the Hidden Markov Model (HMM) profile of the WRKY domain (PF03106) was retrieved from the Pfam database (https://pfam.xfam.org/, accessed on 25 August 2025) and used for initial identification of WRKY candidates using HMMER 3.0 software with a threshold E-value < 1 × 10^−5^. Redundant sequences were manually removed after verification. Candidate WRKY sequences were further validated for the integrity of conserved domains using Pfam and the NCBI Conserved Domain Database (CDD; https://www.ncbi.nlm.nih.gov/Structure/bwrpsb/bwrpsb.cgi, accessed on 25 August 2025). Domain position files were downloaded for subsequent gene structure analysis. A final WRKY dataset for downstream analysis was obtained by aligning these sequences with 72 *Arabidopsis* WRKY protein sequences. Physicochemical properties, including molecular weight, isoelectric point (pI), and protein instability index, were analyzed using the Expasy 3.0 online platform (https://web.expasy.org/, accessed on 26 August 2025). Subcellular localization predictions were performed using WoLF PSORT (https://wolfpsort.hgc.jp/, accessed on 25 August 2025).

### 2.2. Gene Structure and Conserved Motif Analysis of WRKY Transcription Factors

The conserved domains of the WRKY transcription factor family in *D. odorifera* were analyzed using the built-in MEME Suite function in TBtools software v1.2.310 to investigate motif distribution, arrangement, and conservation. Gene annotation files were uploaded to the online platform (https://meme-suite.org/meme/tools/meme, accessed on 1 September 2025) for alignment and analysis, with the number of conserved motifs searched set to 10 and other parameters retained as defaults. The Gene Structure View function of TBtools was employed for simultaneous visualization of exon/intron structures and conserved motifs, enabling integrated structural and functional analysis [20].

### 2.3. Chromosomal Localization and Collinearity Analysis

Chromosomal position information for each *D. odorifera* WRKY gene was obtained from its genome database. To identify whole-genome duplication (WGD), segmental, tandem, proximal, and dispersed duplication events among WRKY family members, One Step MCscanX program of TBtools software v1.2.310 with the default parameters and finally visualized and analyzed on a Circos tool [20,21].

### 2.4. Phylogenetic and Functional Prediction of DodWRKYs

The evolutionary relationships among WRKY family members in *D. odorifera* DodWRKYs were explored using MEGA 7.0 software, with *A. thaliana* WRKY sequences (AtWRKYs) as a reference. A neighbor-joining (NJ) phylogenetic tree was constructed by aligning the full-length WRKY amino acid sequences (94 DodWRKYs and 72 AtWRKYs) using ClustalW. Parameters included Poisson correction, pairwise deletion, and bootstrap analysis with 1000 replicates. The phylogenetic tree was visualized and refined using the Interactive Tree of Life v7 (iTOL) online platform (https://itol.embl.de/, accessed on 2 September 2025). The classification of *D. odorifera* WRKY proteins was determined based on the phylogenetic relationships of *A. thaliana* AtWRKYs. Potential candidate DodWRKY transcription factors involved in heartwood formation and biosynthesis were predicted by inferring their biological functions from the phylogenetic tree.

### 2.5. Cis-Acting Element Analysis of DodWRKY Promoters

The 2000 bp upstream sequences of *D. odorifera* DodWRKY genes were extracted from the genome database and submitted to the PlantCARE website (http://bioinformatics.psb.ugent.be/webtools/plantcare/html/, accessed on 10 September 2025) to predict cis-acting regulatory elements in their promoter regions. Statistical analysis of the identified cis-acting elements was performed, and the results were visualized using TBtools.

### 2.6. Expression of DodWRKY Genes in Different Tissue and Distinct Locations in the Xylem

To elucidate the spatiotemporal expression patterns of the WRKY gene family in *D. odorifera*, we integrated transcriptome data from multiple tissue organs and different developmental stages of xylem. The transcriptional expression profiles of seeds, roots, leaves, and stems were obtained from the published dataset [7], which presented the first chromosome-scale genome assembly of *D. odorifera* based on Illumina paired-end sequencing, Pacific Bioscience single-molecule real-time sequencing, 10× Genomics linked-reads and Hi-C technology, assembled ten super-scaffolds corresponding to the 10 chromosomes with 97.68% of the genome assembled, and also completed gene prediction and functional annotation of *D. odorifera*, providing the first chromosome-level genomic reference resource for its functional gene mining, from which WRKY gene expression data were extracted.

RNA extracted from the outer sapwood, middle sapwood, inner sapwood, outer transition zone, and inner transition zone of the xylem of *D. odorifera* was submitted to Gene Deveo Co., Ltd. (Guangzhou, China) for transcriptome sequencing [22]. Sequence alignment was performed, and the transcriptional expression of the *DodWRKY* gene in each sample was statistically analyzed. Gene expression levels were calculated using the FPKM (Fragments Per Kilobase of exon model per Million mapped fragments) method. To visually represent the expression characteristics of this gene family, hierarchical clustering heatmaps were generated using the online tools provided by the MetWare Bioinformatics Platform (https://www.metware.cn/, accessed on 20 September 2025) [23].

## 3. Results

### 3.1. Identification and Physicochemical Characterization of WRKY Gene Family Members in D. odorifera

Using the Hidden Markov Model (PF03106) as a query, WRKY family members containing the conserved WRKYGQK(E)K domain were screened from the *D. odorifera* genome. After removing duplicates, a total of 94 WRKY family members were identified and named *DodWRKY1* to *DodWRKY94* based on their chromosomal locations (Supplementary Table S1). The physicochemical properties of these 94 DodWRKY transcription factors were analyzed. Results revealed significant variations in amino acid length, isoelectric point (pI), molecular weight, and instability index (Supplementary Table S1). The amino acid lengths of DodWRKY proteins ranged from 142 aa (DodWRKY42) to 763 aa (DodWRKY3), with corresponding molecular weights varying between 16,701.74 and 83,167.12 Da. The instability indices ranged from 36.9 to 70.39, with only four proteins (DodWRKY23, DodWRKY48, DodWRKY79, and DodWRKY81) classified as stable (instability index < 40), indicating that the majority of *D. odorifera* WRKY transcription factors are unstable and prone to degradation. The pI values of DodWRKY proteins spanned from 4.81 to 9.90, with 56 members being acidic (pI < 7.0) and 38 members basic (pI > 7.0), suggesting functional diversity among these proteins. Hydrophobicity analysis predicted that all DodWRKY proteins are hydrophilic (average hydrophilicity index < 0). Subcellular localization predictions indicated that DodWRKY29 and DodWRKY62 localize to peroxisomes, DodWRKY40 to the cytoplasm, DodWRKY22 to chloroplasts, and DodWRKY71 to the plasma membrane. The remaining 89 members (94.68%) were predominantly nuclear-localized. The substantial heterogeneity in amino acid length, molecular weight, pI, and instability index among WRKY family members (Supplementary Table S1) underscores their diverse roles in plant physiological processes.

### 3.2. Evolutionary Analysis of the WRKY Gene Family in D. odorifera

**Figure 1:**
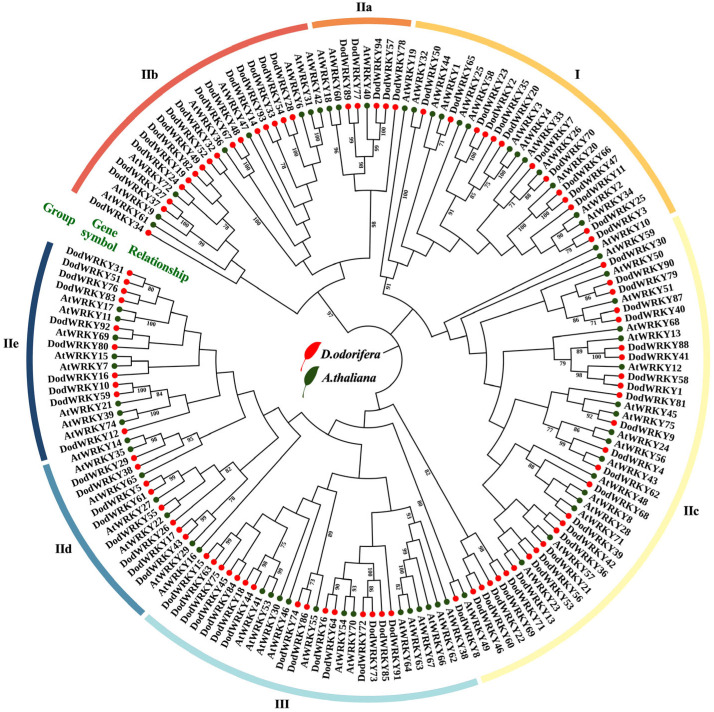
Phylogenetic tree of WRKY transcription factor families in *D. odorifera* and *A. thaliana*. Note: Based on the phylogenetic relationships of WRKY proteins from *D. odorifera* and *A. thaliana*, the tree is divided into three major clades: Group I, II, and III. Group II is further subdivided into five subgroups (IIa, IIb, IIc, IId, and IIe). Red circles represent WRKY proteins from *D. odorifera*, while green circles denote those from *A. thaliana*.

To elucidate the phylogenetic relationships and evolutionary classification of the WRKY gene family in *D. odorifera*, a phylogenetic tree was constructed based on 94 WRKY protein sequences from *D. odorifera* and 72 well-annotated WRKY protein sequences from *A. thaliana* (Figure 1). Phylogenetic analysis demonstrated that the evolutionary classification of *D. odorifera* WRKY transcription factors aligns with the established phylogenetic framework of the *Arabidopsis* WRKY family [20], categorizing them into three major evolutionary clades. Group I, the smallest clade, comprises 13 members (13.8% of the total), characterized predominantly by the presence of two WRKY conserved domains. Notably, however, the DodWRKY23 protein in this group exhibits only a single WRKY domain (Supplementary Figure S1). Group III consists of 15 genes (16.0%). Group II, the largest cluster, contains 66 members (70.2%) and is further subdivided into five subgroups (IIa, IIb, IIc, IId, and IIe) following the *Arabidopsis* classification system. Among these, subgroup IIc represents the predominant branch with 21 members (31.8% of Group II), followed by subgroup IIb with 16 members (24.2%).

### 3.3. Analysis of Gene Structures and Conserved Domains of WRKY Gene Family in D. odorifera

The exon/intron structure provides comprehensive insights into the diversity of gene structures and facilitates the understanding of evolutionary relationships within gene families [4].

**Figure 2:**
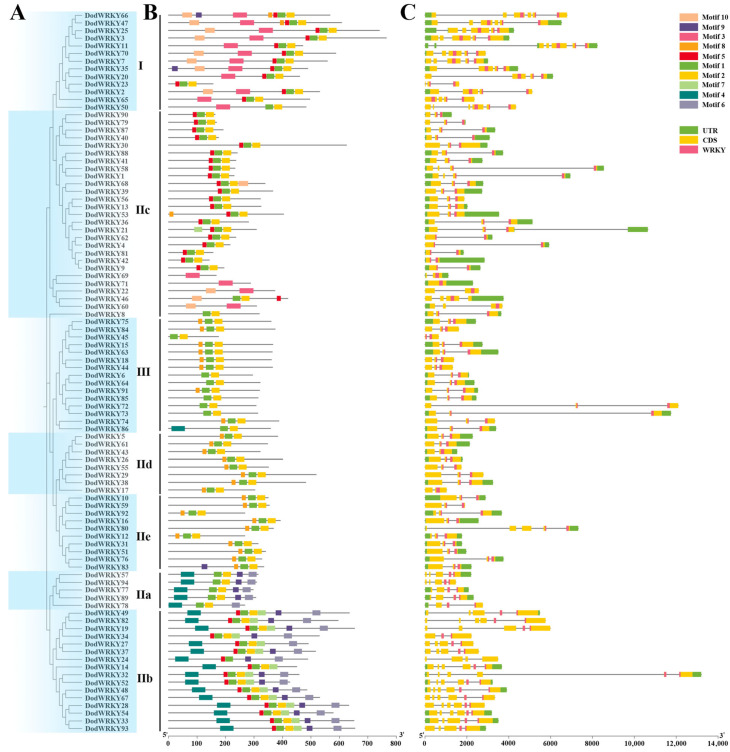
Motif sequence and gene structure of WRKY in *D. odorifera*. (**A**) Phylogenetic tree built using 94 *D. odorifera* WRKY proteins. (**B**) Architectures of conserved protein motifs. The different colored boxes indicate distinct motifs and their corresponding positions in each WRKY protein sequence. The detailed characteristics of each motif are shown in Figure S3. (**C**) Exon/intron structures of *D. odorifera WRKYs*.

In this study, we systematically characterized the structural features of the WRKY gene family in *D. odorifera* (Figure 2). The analysis revealed that, except for *DodWRKY69*, which lacks introns, the remaining 93 WRKY genes contain 1–6 introns. Among these, 46 (48.9%), 12 (12.8%), and 13 (13.8%) genes possess 2, 3, and 4 introns, respectively. Although the number of exons and introns varies, members within the same subgroup exhibit significant structural conservation: Group I genes contain 3–7 exons, Group II genes predominantly have 3 exons, and Group III genes mostly consist of 5 exons.

Conserved motif analysis of the 94 WRKY genes was performed, revealing 10 characteristic conserved motifs (Motif1–Motif10) with amino acid lengths ranging from 15 to 50 (Figure 2; Supplementary Figure S2). Notably, Motif1 and Motif3 correspond to the WRKYGQK domain, the core motif of the WRKY transcription factor family, while Motif2 represents the zinc finger structure. Most WRKY family members in *D. odorifera* contain Motif1, Motif2, or Motif3. However, three transcription factors (*WRKY22*, *WRKY69*, and *WRKY71*) in Subgroup IIc lack Motif1 and Motif2 but retain Motif3. The remaining 91 WRKY transcription factors harbor both Motif1 and Motif2. Within the same subgroup, most members share one or more identical motifs outside the WRKY domain, suggesting potential functional conservation. In contrast, significant differences in motif composition across subgroups provide structural biological evidence for functional divergence among *WRKY* genes. Overall, the motif distribution patterns align closely with the phylogenetic topology, further validating the molecular phylogenetic basis for subgroup classification.

### 3.4. Analysis of Cis-Acting Elements in the Promoters of DodWRKY Genes in D. odorifera

**Figure 3:**
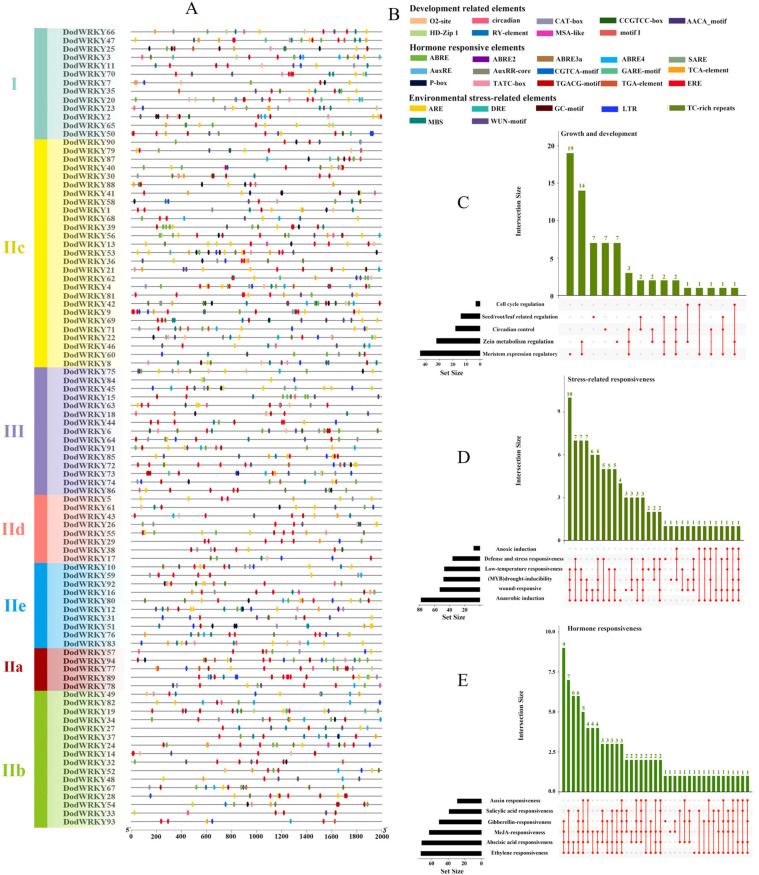
Functional analysis of cis-regulatory elements in the promoters of WRKY genes from *D. odorifera*. Note: (**A**) Positional distribution of cis-acting elements associated with development, hormone response, and environmental stress; (**B**) Functional classification and annotation of cis-acting elements; (**C**) Functional categorization and quantitative distribution of growth- and development-related regulatory elements in promoters; (**D**) Functional classification and distribution frequency of abiotic stress-responsive elements in promoters; (**E**) Functional categorization and abundance of plant hormone signaling-responsive elements in promoters.

Cis-acting elements in promoter regions influence gene expression. Using PlantCARE software, a systematic analysis of the 2000 bp upstream sequences from the transcription start sites of *D. odorifera* WRKY genes identified 102 cis-acting elements with biological functions (Supplementary Table S2). The promoter regions of *DodWRKY* genes harbor diverse regulatory elements, suggesting a complex and multifunctional regulatory network governing their expression. These elements were classified into functional categories, including light-responsive, development-related, hormone-responsive, and environmental stress-related elements (Figure 3). Development-related elements included those involved in cell cycle regulation (distributed in 4 DodWRKY genes), circadian control (18 genes), meristem expression regulation (44 genes), seed/root/leaf-related regulation (14 genes), and zein metabolism regulation (32 genes). Hormone-responsive elements comprised 15 types, participating in responses to abscisic acid (72 genes), auxin (72 genes), salicylic acid (40 genes), gibberellin (51 genes), Methyl Jasmonate (64 genes), and ethylene (73 genes). Environmental stress-related elements included those associated with zein metabolism regulation (78 genes), anoxic induction (8 genes), defense and stress responsiveness (36 genes), MYB-binding drought-inducibility (48 genes), low-temperature responsiveness (47 genes), and wound responsiveness (53 genes). These findings suggest that abiotic stresses, such as drought, low temperature, anoxia, wounding, and defense responses, are closely linked to WRKY gene expression. This study reveals the multidimensional regulatory potential of *DodWRKY* genes in plant growth and development, hormone signaling, and environmental adaptation, providing a critical foundation for deciphering their biological functions through cis-regulatory networks.

### 3.5. Chromosomal Distribution and Collinearity Analysis of DodWRKYs

**Figure 4:**
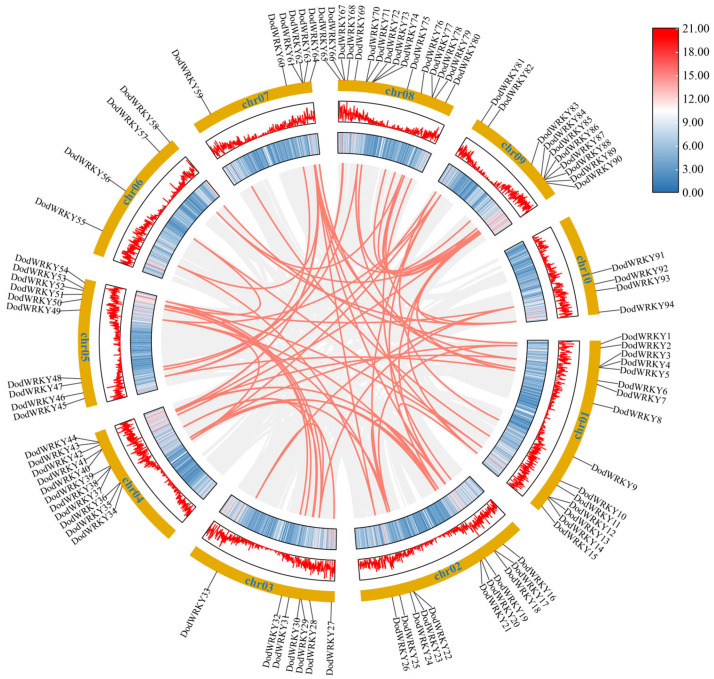
Schematic representations of collinear relationships and location of *D. odorifera WRKYs* on chromosomes. Gray and red lines represent all synteny blocks and duplicated *WRKY* gene pairs in the *D. odorifera* genome, respectively. The corresponding number of each chromosome is shown above the chromosome.

The chromosomal distribution of *WRKY* genes was determined using the *D. odorifera* genome GFF annotation file (Figure 4). A total of 94 genes were unevenly distributed across 10 chromosomes, with significant enrichment observed on Chr08 (16 members) and Chr01 (15 members), while Chr06 and Chr10 contained the fewest members (4 each). Notably, even within the same chromosome, the distribution of transcription factors was non-uniform. Most *DodWRKY* genes tended to cluster in specific chromosomal regions, particularly near the ends of chromosomes, whereas the central arm regions exhibited low-density distribution characteristics. This non-random distribution pattern may be associated with uneven chromosomal replication events in *D. odorifera*.

Whole-genome duplication analysis revealed that segmental duplication, tandem duplication, and transposon events collectively contributed to the expansion of the DodWRKY gene family. Collinearity analysis identified 72 collinear gene pairs involving 78 family members (82.98% of the total). Notably, whole-genome or segmental duplication served as the primary driver for the expansion of the WRKY gene family in *D. odorifera*, playing a critical role in its evolutionary history. Additionally, intrachromosomal duplication events were detected, including two tandem duplication events on Chr08. To elucidate the evolutionary selection pressure acting on *DodWRKY* genes, nonsynonymous and synonymous substitution rates (Ka and Ks) were calculated for collinear gene pairs (Supplementary Table S3). The results demonstrated that nearly all collinear gene pairs exhibited a higher ratio of synonymous to nonsynonymous substitutions (Ka/Ks < 1), indicating that purifying selection has dominated the evolution of WRKY genes in *D. odorifera*.

### 3.6. Expression Patterns of DodWRKY Genes in Different Tissues of D. odorifera

Based on transcriptome sequencing data from four tissues/organs (root, leaf, seed, and stem) of *D. odorifera*, in which the root was further divided into five distinct xylem subregions, this study systematically investigated the expression patterns and potential molecular functions of *DodWRKYs* across different tissues. Most *DodWRKY* genes exhibited low expression abundance in all tissues, with two genes undetectable (FPKM = 0) in all examined tissues (root, leaf, seed, and stem; Supplementary Table S4). Hierarchical clustering analysis categorized the remaining 92 family members into four clusters (Figure S3), demonstrating significant tissue-specific expression patterns. Among these, 46 genes (Cluster IV) were predominantly expressed in roots, while 13 members (Cluster I) and 17 members (Cluster III) showed preferential high expression in leaves and seeds, respectively.

**Figure 5:**
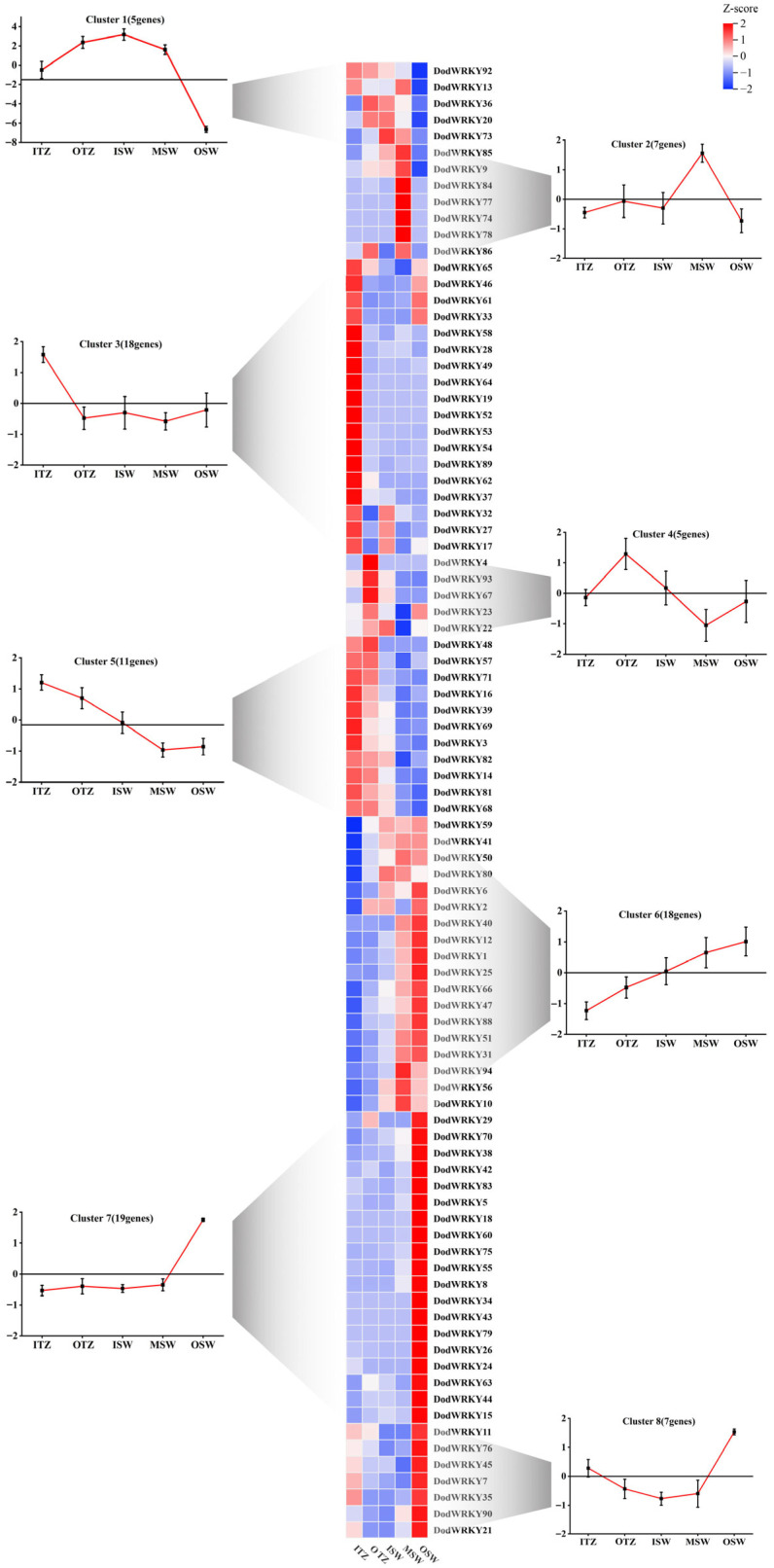
Expression patterns of WRKY TFs in different parts of the xylem clustered in 7 expression groups. Transcript abundance is expressed in z-score standardized fragments per kilobase of exon per million fragments mapped (FPKM) values. For each gene, its name is shown to the right of the heatmap, whereas the short name of the phylogenetic subgroup is included to the left. Next to each RNA seq-based cluster there is a graph with the mean transcript abundance ± SD for the entire cluster.

Transcriptional profiling of xylem subregions (outer sapwood—middle sapwood—inner sapwood—outer transition zone—inner transition zone) revealed differential expression patterns of 90 *DodWRKY* genes associated with economically valuable wood formation (Supplementary Table S5). These genes were classified into eight distinct expression clusters (Figure 5). Cluster 1 (5 genes) exhibited consistently low expression levels, reaching minimal values in outer xylem regions. The majority of *DodWRKYs* (51 genes) were distributed across Clusters 2, 6, 7, and 8. Notably, Cluster 7 members showed specific high expression in outer xylem, while Cluster 2 demonstrated sapwood-specific expression patterns. Thirty-two genes displayed significant upregulation in the xylem transition zone (Clusters 3–5). Cluster 4 (5 genes) maintained low transcriptional activity (FPKM < 15) despite moderate expression in the outer transition zone. Cluster 3 (18 genes) exhibited specific high expression in the inner transition zone, with *DodWRKY58* showing dominant expression (FPKM > 100) and ≥2-fold upregulation compared to outer sapwood. Cluster 5 (11 genes) displayed upregulated expression in both transition subregions, with *DodWRKY14* and *DodWRKY68* identified as differentially expressed genes maintaining high expression levels (FPKM > 50). The spatiotemporal correlation between *DodWRKYs’* specific expression and heartwood formation suggests that transition zone-enriched genes (*DodWRKY58*, *DodWRKY14*, and *DodWRKY68*) may be involved in critical biological processes in xylem secondary metabolite synthesis and heartwood development.

## 4. Discussion

The heartwood of *D. odorifera* holds significant application value in high-end furniture manufacturing, handicraft processing, and traditional Chinese medicine due to its high density, distinctive aromatic characteristics, and abundant medicinal bioactive components [2]. Given its exceptional utility and economic importance, it has garnered extensive attention in recent years [24,25]. As critical regulatory factors, the WRKY transcription factor family plays pivotal roles in plant secondary metabolism regulation, growth and development, and stress responses [26]. Leveraging the newly released whole-genome data of *D. odorifera*, this study systematically identified WRKY family members in this species for the first time, providing crucial theoretical foundations for elucidating heartwood formation mechanisms and regulatory networks of secondary metabolite biosynthesis. Notable variation exists in WRKY family sizes across plant species, reflecting differential expansion events during evolutionary processes. Representative species include *A*. *thaliana* (76 members) [27], *P*. *sativum* (89 members) [11], and *Cannabis sativa* (48 members) [28]. However, systematic investigation of WRKY genes in *D. odorifera* remains unreported. Through genome-wide screening, we successfully identified 94 *DodWRKY* genes. The encoded proteins exhibited substantial diversity in physicochemical properties, including amino acid sequence length (142–763 aa), isoelectric point (4.81–9.9), molecular weight (16.70–83.17 kDa), and instability index (36.9–70.39) (Supplementary Table S1), suggesting functional diversification within this transcription factor family.

Based on the phylogenetic analysis of WRKY transcription factors between *D. odorifera* and *A. thaliana*, this study systematically elucidated the classification and evolutionary relationships of the WRKY gene family in *D. odorifera* (Figure 1). As a model plant, the functional characterization of WRKY transcription factors in *A. thaliana* has been well established. Following the classification criteria of the *Arabidopsis* WRKY family, we categorized the *D. odorifera* WRKY gene family into three major groups (I, II, and III), with Group II further subdivided into five subgroups (IIa-IIe). Notably, the IIc subgroup exhibited the highest number of members (21), consistent with findings in *A. thaliana* [24] and *C*. *sativa* [28], suggesting that the IIc subgroup may have undergone significant gene duplication events during plant evolution. Phylogenetic analysis revealed that Group I members generally retain the characteristic dual WRKY domains (Supplementary Figure S1), whereas the DodWRKY23 protein possesses only a single domain. We hypothesize that this protein likely originated from a dual-domain ancestral gene and experienced domain loss during evolution, a phenomenon similarly observed in *Chrysanthemum lavandulifolium* (Asteraceae) and *Salix suchowensis* (Salicaceae) [29,30]. Genes with analogous biological functions often cluster within the same subgroup, providing critical insights into their roles in plant growth and development [31]. For instance, *AtWRKY33* in *A. thaliana* plays a pivotal role in plant immune regulation. Its ortholog, *DodWRKY7*, displays high expression levels in stem tissues and clusters within the same phylogenetic clade, suggesting potential functional importance in xylem development or stem immune defense mechanisms [32].

Chromosomal localization analysis revealed that *DodWRKY* genes are clustered heterogeneously across chromosomes in *D. odorifera*, potentially resulting from uneven duplication events of chromosomal fragments (Figure 4). Segmental and tandem duplications were identified as primary mechanisms driving the evolutionary expansion and diversification of the WRKY gene family. Collinearity analysis identified two tandem-duplicated WRKY gene pairs on chromosome 8. Furthermore, 70 segmental duplication pairs involving 76 genes were detected across ten chromosomes (Figure 4; Table S3), indicating that segmental duplication played the most significant role in WRKY family expansion in *D. odorifera*. Duplicated transcription factors (TFs) exhibited conserved gene structures and motif compositions, with phylogenetically clustered WRKY TFs showing collinearity relationships. For instance, DodWRKY1 and DodWRKY58, both belonging to subgroup IIc, demonstrated collinearity. These findings suggest that the expansion of the WRKY gene family in *D. odorifera* likely facilitated functional diversification, enhancing environmental adaptability. Introns, critical structural components of eukaryotic genes with multifunctional roles, contribute to structural diversity and complexity through deletion or modification. The predominant intron-exon architecture in *D. odorifera* WRKY genes comprises three exons and two introns (Figure 2). Conserved gene structures within phylogenetic groups may reflect duplication events, implying that divergent exon-intron patterns in WRKY domains might have driven evolutionary diversification. Collectively, gene duplication events expanded the WRKY family while generating structural diversity and functional divergence, though conserved architectures within subgroups corroborate phylogenetic relationships. Cis-acting elements in promoter regions serve as critical regulatory components for gene transcription in plants [33]. To investigate transcriptional regulation mechanisms of *DodWRKY* genes, we analyzed 2000 bp promoter sequences upstream of translation initiation sites. Prediction results revealed that WRKY TFs in *D. odorifera* contain numerous cis-elements including CAT-box, ERE, and DRE (Figure 3), suggesting their involvement in diverse biological processes such as growth regulation, hormonal signaling, and biotic/abiotic stress responses through direct or indirect pathways.

As a perennial woody plant, the primary economic value of *D. odorifera* lies in its xylem, particularly the heartwood. The formation of heartwood in *D. odorifera* involves programmed cell death (PCD) of parenchyma cells in the sapwood and the specific accumulation of secondary metabolites [34,35]. To elucidate the expression patterns of WRKY genes during heartwood formation, this study partitioned the xylem into five regions for transcriptomic analysis: outer sapwood (OSW), middle sapwood (MSW), inner sapwood (ISW), intermediate transition zone (OTZ), and inner transition zone (ITZ) (Figure 5). In mature sapwood regions (OSW-ISW), vessel and fiber cells have completed their morphological development and primarily function in water transport and mechanical support [36]. Numerous *DodWRKYs* exhibited high expression levels in the sapwood, where metabolically active cells maintain primary metabolism and stress defense mechanisms. Among these, 19 highly expressed *DodWRKYs* (e.g., members of Cluster 7) may participate in sustaining primary metabolic homeostasis and stress defense in parenchyma cells. For instance, the specific expression of *DodWRKY44* (homologous to the *Arabidopsis* group III WRKY transcription factor AtWRKY41) in the outer sapwood suggests its potential role in regulating plant immune-related pathways [37], thereby maintaining the physiological activity of sapwood cells.

The transition zone marks critical changes in parenchyma cell physiology closely associated with heartwood formation [38]. Three *DodWRKY* genes showed differential expression and elevated transcript levels (FPKM > 50) in this region. *DodWRKY14*, classified as Group IIb and homologous to AtWRKY6 (known for its involvement in plant defense responses and senescence) [39], was notably upregulated. The largest WRKY subfamily in *D. odorifera*, Group IIc, included *DodWRKY58/68*, which were upregulated in the transition zone. Plant Group IIc WRKY members are implicated in metabolic and defense processes, such as *Arabidopsis* AtWRKY23, which regulates flavonol biosynthesis [40], and TRANSPARENT TESTA GLABRA2 (AtWRKY44), which modulates tannin accumulation [41]. Given the substantial deposition of flavonoids and other secondary metabolites in *D. odorifera* heartwood, Group IIc members likely contribute to this process. Collectively, the spatiotemporal expression patterns of WRKY transcription factors in *D. odorifera* xylem development provide critical insights into the molecular regulatory mechanisms underlying heartwood formation. Future studies will focus on functional validation of key candidate genes (e.g., *DodWRKY14*, *DodWRKY58*, *DodWRKY68*) and elucidation of their roles in regulating secondary metabolic pathways.

## 5. Conclusions

Based on whole-genome data of *D. odorifera*, this study conducted a systematic investigation of the WRKY transcription factor family. Through bioinformatic approaches, 94 DodWRKY transcription factors were identified, exhibiting non-uniform clustered distribution patterns across 10 chromosomes. Genomic collinearity analysis revealed 72 *WRKY* homologous gene pairs, with segmental duplication events identified as the primary mechanism driving the expansion of *DodWRKY* gene family, a process predominantly governed by purifying selection. Phylogenetic analysis classified DodWRKY members into seven canonical subgroups. Three genes from subgroups IIb and IIc (*DodWRKY14*, *DodWRKY58*, and *DodWRKY68*) demonstrated specific expression in the xylem transition zone. These genes potentially regulate heartwood formation through modulation of secondary metabolite biosynthesis pathways.

## Data Availability

The original contributions presented in the study are included in the article/Supplementary Materials. Further inquiries can be directed to the corresponding authors.

## Supplementary Materials

The following supporting information can be downloaded at: https://www.mdpi.com/article/10.3390/genes17040386/s1, Figure S1: Amino acid sequence alignment of conserved domains in DodWRKY and AtWRKY. “N” and “C” denote the N-terminus and C-terminus of the conserved domains in WRKY proteins, respectively; Figure S2: Conserved motifs of WRKY transcription factors; Figure S3: Expression patterns of WRKY transcription factors in different tissues (root, stem, leaf, and seed) of *Dalbergia odorifera*. Note: Transcript abundance is represented by z-score normalized RPKM values; Table S1: Detailed characteristics of the WRKY proteins in this study; Table S2: Cis-element analysis of promoter regions of *DodWRKY* genes; Table S3: Tandem and segmental duplication events of *WRKYs* members in *D. odorifera*; Table S4: Transcript expression of *WRKYs* in different tissue parts of *D. odorifera*; Table S5: Transcript expression of WRKYs in different tissue parts of *D. odorifera* xylem.
