# Supplementary material for: Genome-Wide Characterization and Transcriptional Profiling of the WRKY Gene Family During Heartwood Formation in Dalbergia odorifera
Source: Genes (Basel). 2026 Mar 28;17(4):386. doi: 10.3390/genes17040386 (PMC13116284; doi:10.3390/genes17040386)
Supplement: Supplementary file 1 [file genes-17-00386-s001.zip › Figure S1-3.pdf]

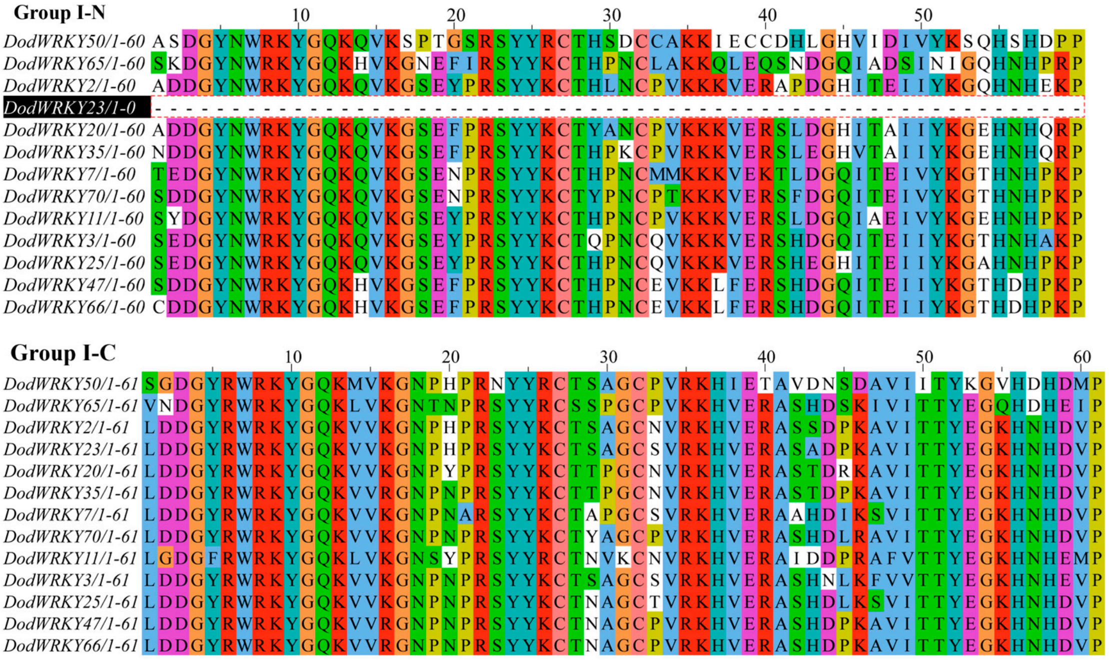

Figure S1. Amino acid sequence alignment of conserved domains in DodWRKY and AtWRKY. "N" and "C" denote the N-terminus and C-terminus of the conserved domains in WRKY proteins, respectively.

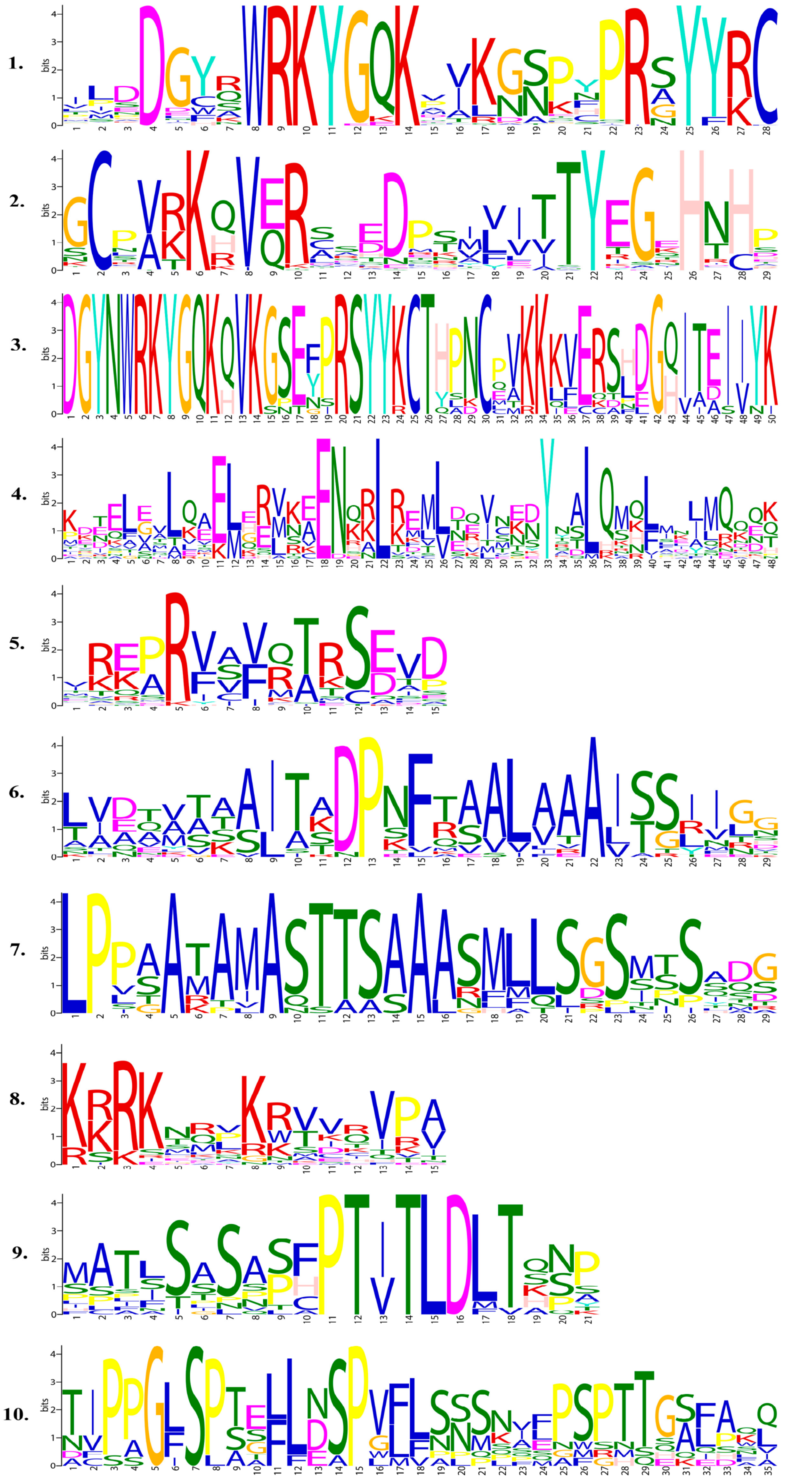

Figure S2. Conserved motifs of WRKY transcription factors.

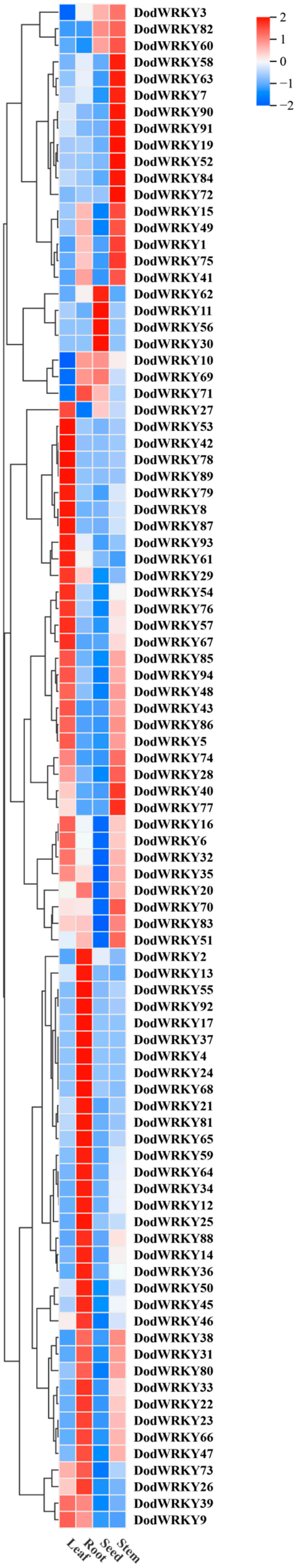

Figure S3. Expression patterns of WRKY transcription factors in different tissues (root, stem, leaf, and seed) of *Dalbergia odorifera*. Note: Transcript abundance is represented by z-score normalized RPKM values.
